# Supplementary material for: Effects of non-pharmacological interventions on cognitive function in patients with type 2 diabetes mellitus and mild cognitive impairment: A network meta-analysis
Source: PLoS One. 2025 Aug 12;20(8):e0329397. doi: 10.1371/journal.pone.0329397 (PMC12342316; doi:10.1371/journal.pone.0329397)
Supplement: S1 Table — (PDF) [file pone.0329397.s002.pdf]

Pubmed

#1	"Diabetes Mellitus, Type 2"[Mesh]
#2	((((((((((((((((((((((((((((((Diabetes Mellitus, Adult-Onset[Title/Abstract]) OR (Adult-Onset Diabetes Mellitus[Title/Abstract])) OR (Diabetes Mellitus, Adult Onset[Title/Abstract])) OR (Diabetes Mellitus, Ketosis-Resistant[Title/Abstract])) OR (Diabetes Mellitus, Ketosis Resistant[Title/Abstract])) OR (Ketosis-Resistant Diabetes Mellitus[Title/Abstract])) OR (Diabetes Mellitus, Non Insulin Dependent[Title/Abstract])) OR (Diabetes Mellitus, Non-Insulin-Dependent[Title/Abstract])) OR (Non-Insulin-Dependent Diabetes Mellitus[Title/Abstract])) OR (Diabetes Mellitus, Stable[Title/Abstract])) OR (Stable Diabetes Mellitus[Title/Abstract])) OR (Diabetes Mellitus, Type II[Title/Abstract])) OR (NIDDM[Title/Abstract])) OR (Diabetes Mellitus, Noninsulin Dependent[Title/Abstract])) OR (Diabetes Mellitus, Maturity-Onset[Title/Abstract])) OR (Diabetes Mellitus, Maturity Onset[Title/Abstract])) OR (Maturity-Onset Diabetes Mellitus[Title/Abstract])) OR (Maturity Onset Diabetes Mellitus[Title/Abstract])) OR (MODY[Title/Abstract])) OR (Diabetes Mellitus, Slow-Onset[Title/Abstract])) OR (Diabetes Mellitus, Slow Onset[Title/Abstract])) OR (Slow-Onset Diabetes Mellitus[Title/Abstract])) OR (Type 2 Diabetes Mellitus[Title/Abstract])) OR (Noninsulin-Dependent Diabetes Mellitus[Title/Abstract])) OR (Noninsulin Dependent Diabetes Mellitus[Title/Abstract])) OR (Maturity-Onset Diabetes[Title/Abstract])) OR (Diabetes, Maturity-Onset[Title/Abstract])) OR (Maturity Onset Diabetes[Title/Abstract])) OR (Type 2 Diabetes[Title/Abstract])) OR (Diabetes, Type 2[Title/Abstract])) OR (Diabetes Mellitus, Noninsulin-Dependent[Title/Abstract])
#3	#1 OR #2
#4	"Cognitive Dysfunction"[Mesh]
#5	((((((((((((((((((((((((Cognitive Dysfunctions[Title/Abstract]) OR (Dysfunction, Cognitive[Title/Abstract])) OR (Dysfunctions, Cognitive[Title/Abstract])) OR (Cognitive Disorder[Title/Abstract])) OR (Cognitive Disorders[Title/Abstract])) OR (Disorder, Cognitive[Title/Abstract])) OR (Disorders, Cognitive[Title/Abstract])) OR (Cognitive Impairments[Title/Abstract])) OR (Cognitive Impairment[Title/Abstract])) OR (Impairment, Cognitive[Title/Abstract])) OR (Impairments, Cognitive[Title/Abstract])) OR (Mild Cognitive Impairment[Title/Abstract])) OR (Cognitive Impairment, Mild[Title/Abstract])) OR (Cognitive Impairments, Mild[Title/Abstract])) OR (Impairment, Mild Cognitive[Title/Abstract])) OR (Impairments, Mild Cognitive[Title/Abstract])) OR (Mild Cognitive Impairments[Title/Abstract])) OR (Cognitive Decline[Title/Abstract])) OR (Cognitive Declines[Title/Abstract])) OR (Decline, Cognitive[Title/Abstract])) OR (Declines, Cognitive[Title/Abstract])) OR

	(Mental Deterioration[Title/Abstract])) OR (Deterioration, Mental[Title/Abstract])) OR (Deteriorations, Mental[Title/Abstract])) OR (Mental Deteriorations[Title/Abstract])
#6	#4 OR #5
#7	randomized controlled trial[Publication Type] OR randomized[Title/Abstract] OR placebo[Title/Abstract]
#8	#3 AND #6 AND #7

Embase

#1	'non insulin dependent diabetes mellitus'/exp
#2	'adult onset diabetes':ab,ti OR 'adult onset diabetes mellitus':ab,ti OR 'diabetes mellitus type 2':ab,ti OR 'diabetes mellitus type ii':ab,ti OR 'diabetes mellitus, maturity onset':ab,ti OR 'diabetes mellitus, non insulin dependent':ab,ti OR 'diabetes mellitus, non-insulin-dependent':ab,ti OR 'diabetes mellitus, type 2':ab,ti OR 'diabetes mellitus, type ii':ab,ti OR 'diabetes type 2':ab,ti OR 'diabetes type ii':ab,ti OR 'diabetes, adult onset':ab,ti OR 'dm 2':ab,ti OR 'insulin independent diabetes':ab,ti OR 'insulin independent diabetes mellitus':ab,ti OR 'ketosis resistant diabetes mellitus':ab,ti OR 'maturity onset diabetes':ab,ti OR 'maturity onset diabetes mellitus':ab,ti OR 'niddm':ab,ti OR 'niddm (non insulin dependent diabetes mellitus)':ab,ti OR 'non insulin dependent (type 2) diabetes mellitus':ab,ti OR 'non insulin dependent diabetes':ab,ti OR 'non-insulin-dependent diabetes mellitus':ab,ti OR 'noninsulin dependent (type 2) diabetes mellitus':ab,ti OR 'noninsulin dependent diabetes':ab,ti OR 'noninsulin dependent diabetes mellitus':ab,ti OR 't2dm':ab,ti OR 'tiidm':ab,ti OR 'type 2 (insulin independent) diabetes':ab,ti OR 'type 2 diabetes':ab,ti OR 'type 2 diabetes mellitus':ab,ti OR 'type ii diabetes':ab,ti OR 'type ii diabetes mellitus':ab,ti OR 'non insulin dependent diabetes mellitus':ab,ti
#3	#1 OR #2
#4	'cognitive defect'/exp
#5	'cognition disorder':ab,ti OR 'cognition disorders':ab,ti OR 'cognitive complaints':ab,ti OR 'cognitive decline':ab,ti OR 'cognitive defects':ab,ti OR 'cognitive deficiency':ab,ti OR 'cognitive deficit':ab,ti OR 'cognitive difficulties':ab,ti OR 'cognitive disability':ab,ti OR 'cognitive disorder':ab,ti OR 'cognitive disorders':ab,ti OR 'cognitive disturbance':ab,ti OR 'cognitive dysfunction':ab,ti OR 'cognitive impairment':ab,ti OR 'cognitive problems':ab,ti OR 'delirium, dementia, amnestic, cognitive disorders':ab,ti OR 'overinclusion':ab,ti OR 'response interference':ab,ti OR 'cognitive defect':ab,ti
#6	#4 OR #5
#7	'random':ab,ti
#8	'placebo':ab,ti
#9	'double-blind':ab,ti
#10	#7 OR #8 OR #9
#11	#3 AND #6 AND #10

Web of science

1	TS=(Diabetes Mellitus, Type 2) OR TS=(Diabetes Mellitus, Adult-Onset) OR TS=(Adult-Onset Diabetes Mellitus) OR TS=(Diabetes Mellitus, Adult Onset) OR TS=(Diabetes Mellitus, Ketosis-Resistant) OR TS=(Diabetes Mellitus, Ketosis Resistant) OR TS=(Ketosis-Resistant Diabetes Mellitus) OR TS=(Diabetes Mellitus, Non Insulin Dependent) OR TS=(Diabetes Mellitus, Non-Insulin-Dependent) OR TS=(Non-Insulin-Dependent Diabetes Mellitus) OR TS=(Diabetes Mellitus, Stable) OR TS=(Stable Diabetes Mellitus) OR TS=(Diabetes Mellitus, Type II) OR TS=(NIDDM) OR TS=(Diabetes Mellitus, Noninsulin Dependent) OR TS=(Diabetes Mellitus, Maturity-Onset) OR TS=(Diabetes Mellitus, Maturity Onset) OR TS=(Maturity-Onset Diabetes Mellitus) OR TS=(Maturity Onset Diabetes Mellitus) OR TS=(MODY) OR TS=(Diabetes Mellitus, Slow-Onset) OR TS=(Diabetes Mellitus, Slow Onset) OR TS=(Slow-Onset Diabetes Mellitus) OR TS=(Type 2 Diabetes Mellitus) OR TS=(Noninsulin-Dependent Diabetes Mellitus) OR TS=(Noninsulin Dependent Diabetes Mellitus) OR TS=(Maturity-Onset Diabetes) OR TS=(Diabetes, Maturity-Onset) OR TS=(Maturity Onset Diabetes) OR TS=(Type 2 Diabetes) OR TS=(Diabetes, Type 2) OR TS=(Diabetes Mellitus, Noninsulin-Dependent)
2	TS=(Cognitive Dysfunction) OR TS=(Cognitive Dysfunctions) OR TS=(Dysfunction, Cognitive) OR TS=(Dysfunctions, Cognitive) OR TS=(Cognitive Disorder) OR TS=(Cognitive Disorders) OR TS=(Disorder, Cognitive) OR TS=(Disorders, Cognitive) OR TS=(Cognitive Impairments) OR TS=(Cognitive Impairment) OR TS=(Impairment, Cognitive) OR TS=(Impairments, Cognitive) OR TS=(Mild Cognitive Impairment) OR TS=(Cognitive Impairment, Mild) OR TS=(Cognitive Impairments, Mild) OR TS=(Impairment, Mild Cognitive) OR TS=(Impairments, Mild Cognitive) OR TS=(Mild Cognitive Impairments) OR TS=(Cognitive Decline) OR TS=(Cognitive Declines) OR TS=(Decline, Cognitive) OR TS=(Declines, Cognitive) OR TS=(Mental Deterioration) OR TS=(Deterioration, Mental) OR TS=(Deteriorations, Mental) OR TS=(Mental Deteriorations)
3	TS=(randomized controlled trial) OR TS=(randomized) OR TS=(placebo) OR TS=(random) OR TS=(double-blind)
4	#3 AND #2 AND #1

Cochrane library

#1	MeSH descriptor: [Diabetes Mellitus, Type 2] explode all trees
#2	(Diabetes Mellitus, Type 2):ti,ab,kw OR (Diabetes Mellitus, Maturity Onset):ti,ab,kw OR (Ketosis-Resistant Diabetes Mellitus):ti,ab,kw OR (Diabetes, Maturity-Onset):ti,ab,kw OR (Type 2 Diabetes Mellitus):ti,ab,kw
#3	(Diabetes Mellitus, Slow Onset):ti,ab,kw OR (Diabetes Mellitus, Adult-Onset):ti,ab,kw OR (NIDDM):ti,ab,kw OR (Type 2 Diabetes):ti,ab,kw OR (Diabetes Mellitus, Non-Insulin-Dependent):ti,ab,kw

#4	(Diabetes Mellitus, Non Insulin Dependent):ti,ab,kw OR (Diabetes Mellitus, Maturity-Onset):ti,ab,kw OR (Adult-Onset Diabetes Mellitus):ti,ab,kw OR (Diabetes Mellitus, Noninsulin Dependent):ti,ab,kw OR (Diabetes Mellitus, Stable):ti,ab,kw
#5	(Non-Insulin-Dependent Diabetes Mellitus):ti,ab,kw OR (Maturity-Onset Diabetes Mellitus):ti,ab,kw OR (Diabetes Mellitus, Slow-Onset):ti,ab,kw OR (MODY):ti,ab,kw OR (Slow-Onset Diabetes Mellitus):ti,ab,kw
#6	(Maturity Onset Diabetes):ti,ab,kw OR (Maturity-Onset Diabetes):ti,ab,kw OR (Maturity Onset Diabetes Mellitus):ti,ab,kw OR (Stable Diabetes Mellitus):ti,ab,kw OR (Diabetes, Type 2):ti,ab,kw
#7	(Noninsulin-Dependent Diabetes Mellitus):ti,ab,kw OR (Diabetes Mellitus, Ketosis Resistant):ti,ab,kw OR (Noninsulin Dependent Diabetes Mellitus):ti,ab,kw OR (Diabetes Mellitus, Noninsulin-Dependent):ti,ab,kw OR (Diabetes Mellitus, Adult Onset):ti,ab,kw
#8	(Diabetes Mellitus, Ketosis-Resistant):ti,ab,kw OR (Diabetes Mellitus, Type II):ti,ab,kw
#9	#1 OR #2 OR #3 OR #4 OR #5 OR #6 OR #7 OR #8
#10	MeSH descriptor: [Cognitive Dysfunction] explode all trees
#11	(Cognitive Dysfunction):ti,ab,kw OR (Mild Cognitive Impairment):ti,ab,kw OR (Impairments, Mild Cognitive):ti,ab,kw OR (Impairment, Mild Cognitive):ti,ab,kw OR (Mild Cognitive Impairments):ti,ab,kw
#12	(Cognitive Impairments, Mild):ti,ab,kw OR (Cognitive Impairment, Mild):ti,ab,kw OR (Mental Deteriorations):ti,ab,kw OR (Deteriorations, Mental):ti,ab,kw OR (Cognitive Decline):ti,ab,kw
#13	(Decline, Cognitive):ti,ab,kw OR (Mental Deterioration):ti,ab,kw OR (Cognitive Declines):ti,ab,kw OR (Declines, Cognitive):ti,ab,kw OR (Deterioration, Mental):ti,ab,kw
#14	(Disorders, Cognitive):ti,ab,kw OR (Dysfunction, Cognitive):ti,ab,kw OR (Cognitive Disorder):ti,ab,kw OR (Impairments, Cognitive):ti,ab,kw OR (Cognitive Impairments):ti,ab,kw
#15	(Impairment, Cognitive):ti,ab,kw OR (Disorder, Cognitive):ti,ab,kw OR (Cognitive Dysfunctions):ti,ab,kw OR (Cognitive Disorders):ti,ab,kw OR (Cognitive Impairment):ti,ab,kw
#16	(Dysfunctions, Cognitive):ti,ab,kw
#17	#10 OR #11 OR #12 OR #13 OR #14 OR #15 OR #16
#18	#9 AND #17

CNKI

#1	SU=2 型糖尿病 OR SU=二型糖尿病 OR SU=Ⅱ型糖尿病
#2	SU=轻度认知障碍 OR SU=轻度认知损害
#3	#1 AND #2

Wanfang Data

#1	主题:(2 型糖尿病 or 二型糖尿病 or Ⅱ型糖尿病)
#2	主题:(轻度认知障碍 or 轻度认知损害)
#3	#1 AND #2

VIP

#1	主题:(2 型糖尿病 OR 二型糖尿病 OR Ⅱ型糖尿病)
#2	主题:(轻度认知障碍 OR 轻度认知损害)
#3	#1 AND #2

Sinomed

#1	常用字段:(2 型糖尿病 OR 二型糖尿病 OR Ⅱ型糖尿病)
#2	常用字段:(轻度认知障碍 OR 轻度认知损害)
#3	#1 AND #2
