# Supplementary material for: Effects of non-pharmacological interventions on cognitive function in patients with type 2 diabetes mellitus and mild cognitive impairment: A network meta-analysis
Source: PLoS One. 2025 Aug 12;20(8):e0329397. doi: 10.1371/journal.pone.0329397 (PMC12342316; doi:10.1371/journal.pone.0329397)
Supplement: S4 Table — (DOCX) [file pone.0329397.s005.docx]

SUCRA Rank Diagram. a) MoCA, b) MMSE

a)

| Treatment | SUCRA | Rank |
| --- | --- | --- |
| comprehensive intervention | 76.9 | 1 |
| cognitive training | 63.3 | 2 |
| TCM therapy | 57.7 | 3 |
| exercise therapy | 51.7 | 4 |
| usual care | 0.2 | 5 |

b)

| Treatment | SUCRA | Rank |
| --- | --- | --- |
| exercise therapy | 78.0 | 1 |
| cognitive training | 73.8 | 2 |
| TCM therapy | 60.0 | 3 |
| comprehensive intervention | 33.1 | 4 |
| usual care | 5.1 | 5 |
