# Supplementary material for: Effects of non-pharmacological interventions on cognitive function in patients with type 2 diabetes mellitus and mild cognitive impairment: A network meta-analysis
Source: PLoS One. 2025 Aug 12;20(8):e0329397. doi: 10.1371/journal.pone.0329397 (PMC12342316; doi:10.1371/journal.pone.0329397)
Supplement: S5 Table — (DOCX) [file pone.0329397.s006.docx]

**Perform a sensitivity analysis by excluding the study with the smallest sample size and compare it with the heterogeneity before the exclusion.**

Eliminated literature:：Ghodrati N, Haghighi AH, Kakhak SAH, Abbasian S, Gold GS. Effect of Combined Exercise Training on Physical and Cognitive Function in Women With Type 2 Diabetes. Can J Diabetes. 2023 Mar;47(2):162-170. Epub 2022 Nov 17. doi: 10.1016/j.jcjd.2022.11.005. PMID: 36572617.

**
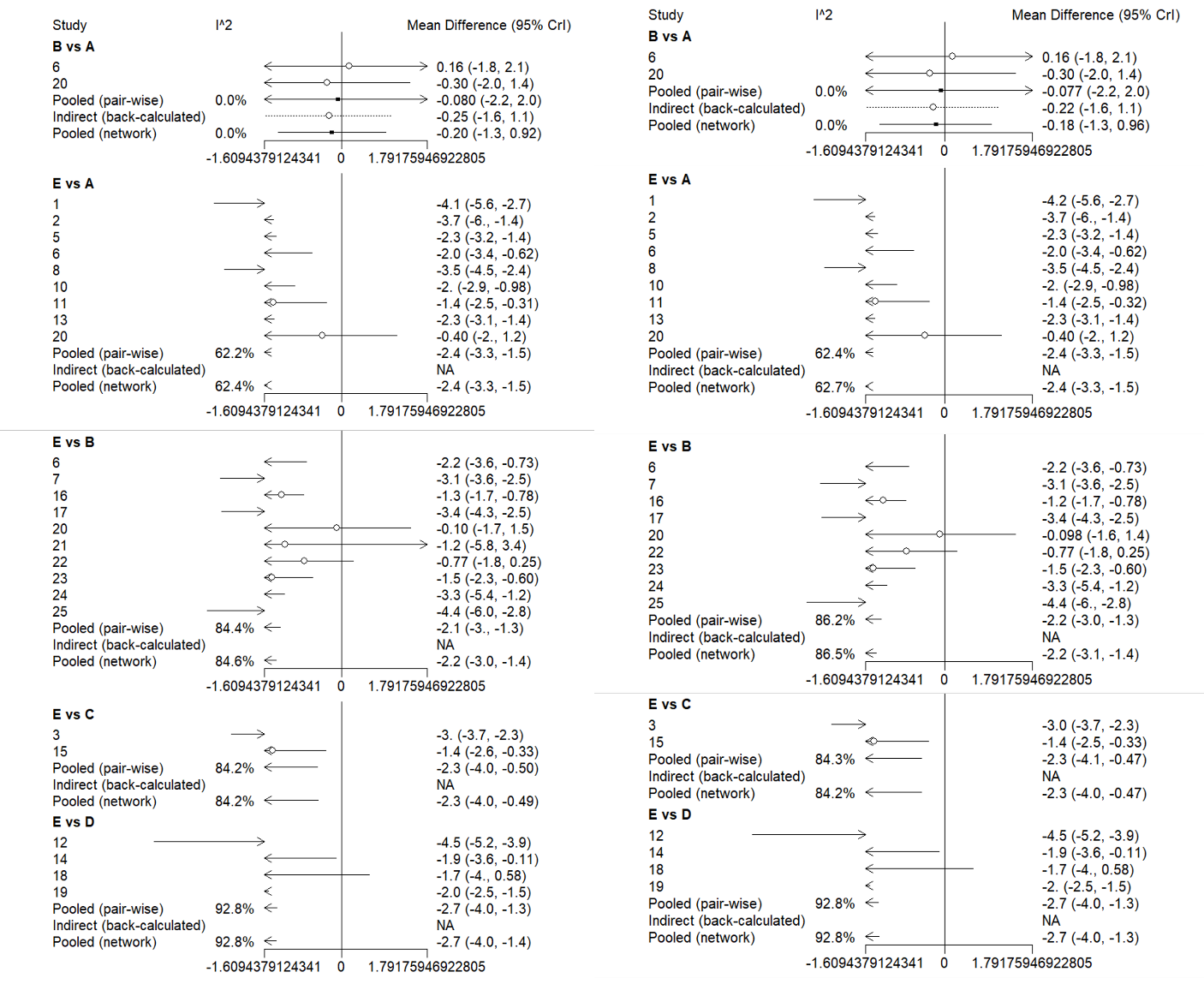
**

Figure 1 The results of heterogeneity tests before and after sensitivity analysis

before(left);after(right)

SUCRA Rank Diagram after sensitivity analysis

| Treatment | SUCRA | Rank |
| --- | --- | --- |
| comprehensive intervention | 76.4 | 1 |
| cognitive training | 63.1 | 2 |
| TCM therapy | 57.5 | 3 |
| exercise therapy | 52.7 | 4 |
| usual care | 0.2 | 5 |


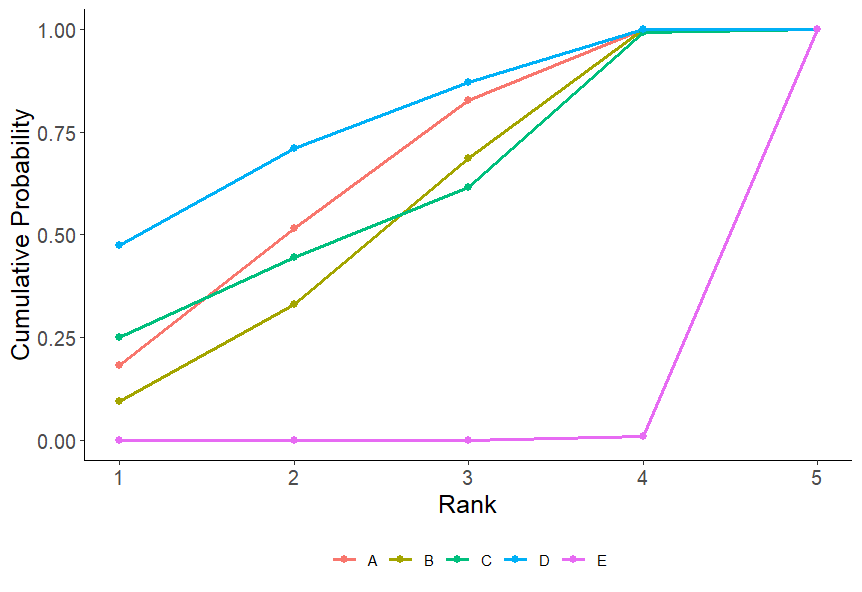


Figure 2 Cumulative probability ranking of MoCA scores for different non-pharmacological intervention measures after sensitivity analysis

Network meta-regression results on MoCA scores

| Covariate | Intervention | B (beta) of comparison | | |
| --- | --- | --- | --- | --- |
|  |  | Mean | 2.5% | 97.5% |
| duration | cognitive training | -0.5193 | -2.2692 | 1.200 |
|  | exercise therapy | -0.2400 | -1.9866 | 1.491 |
|  | TCM therapy | 18.398 | -16.39 | 181.35 |
|  | comprehensive intervention | 24.250 | -35.22 | 152.06 |
| Frequency | cognitive training | 0.1505 | -1.6089 | 1.906 |
|  | exercise therapy | 0.4234 | -1.4050 | 2.278 |
|  | TCM therapy | 0.5926 | -13.5647 | 17.164 |
|  | comprehensive intervention | 1.2660 | -1.4245 | 3.979 |
