# Supplementary material for: Effects of non-pharmacological interventions on cognitive function in patients with type 2 diabetes mellitus and mild cognitive impairment: A network meta-analysis
Source: PLoS One. 2025 Aug 12;20(8):e0329397. doi: 10.1371/journal.pone.0329397 (PMC12342316; doi:10.1371/journal.pone.0329397)
Supplement: S1 Fig — (PDF) [file pone.0329397.s007.pdf]

MoCA

Cognitive training

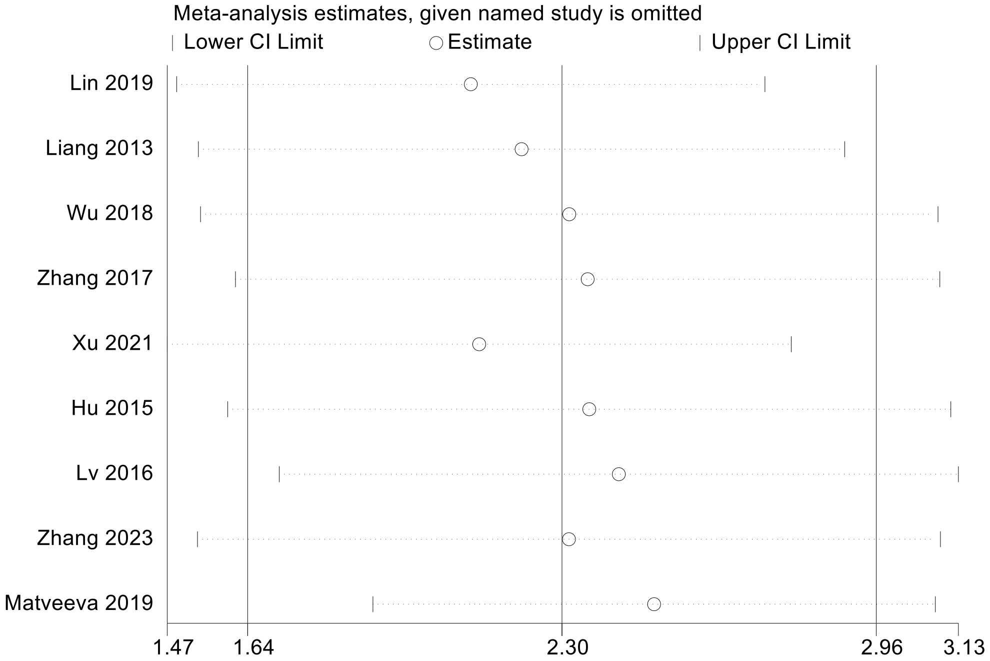

Exercise therapy

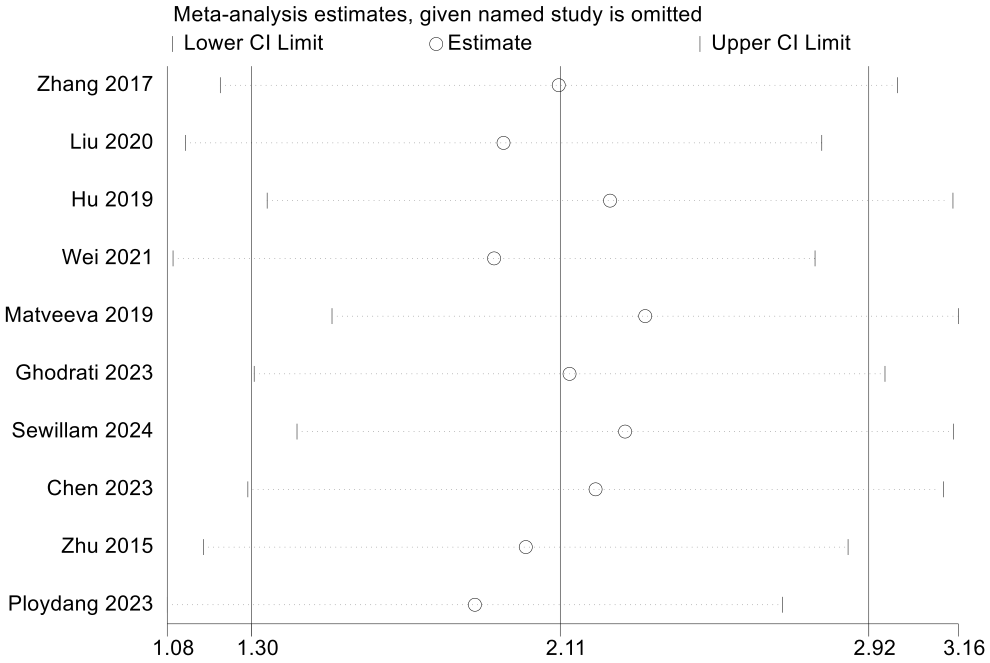

Comprehensive intervention

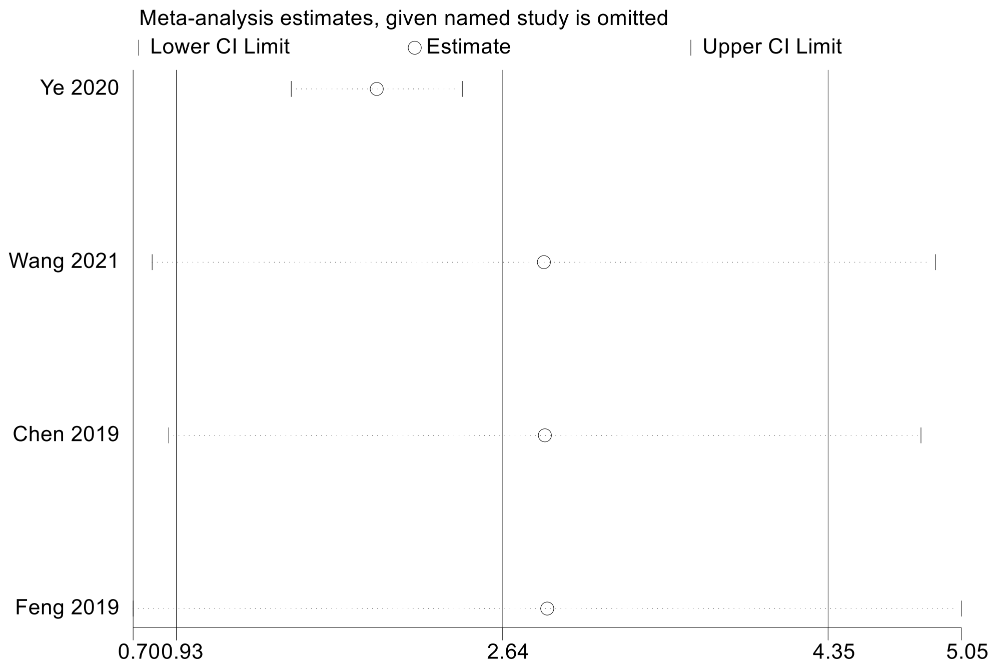

Comprehensive intervention (after excluding studies by Ye 2020)

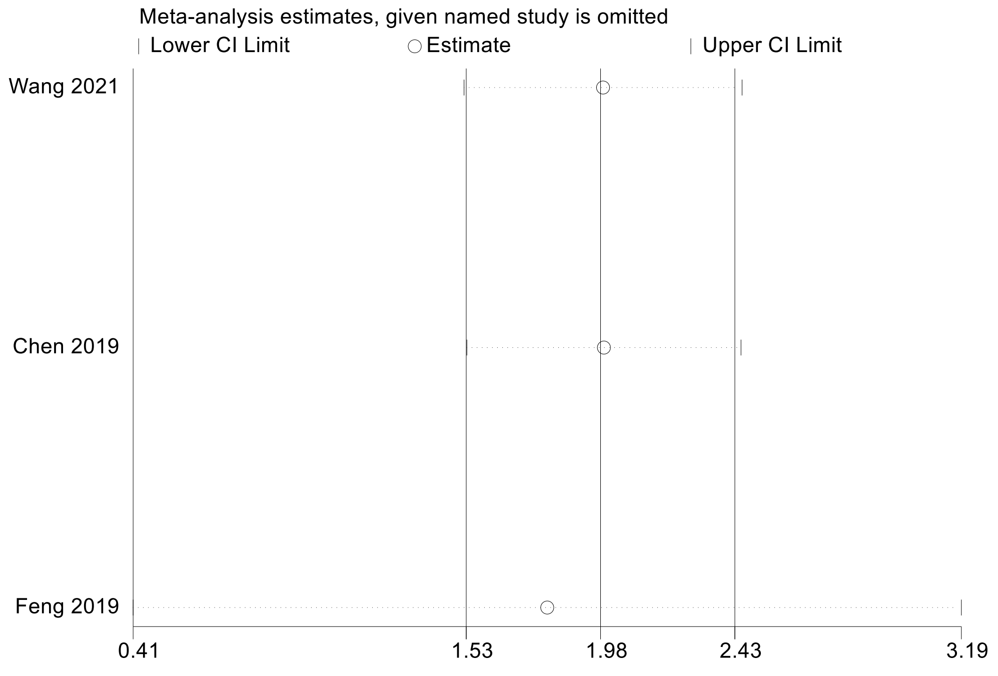

MMSE

Cognitive training

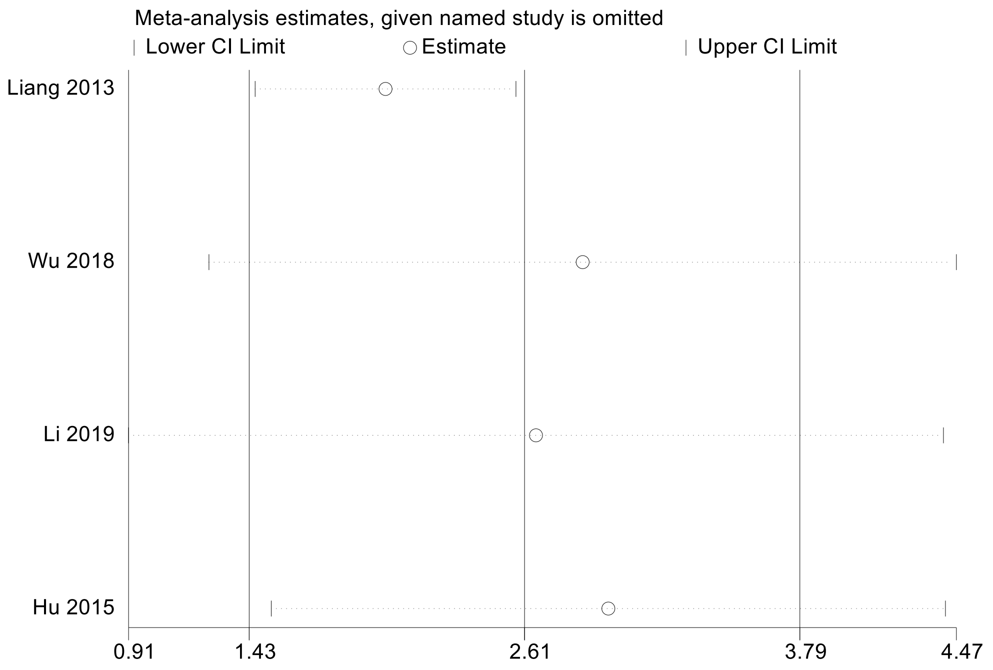

Cognitive training (after excluding studies by Liang 2013)

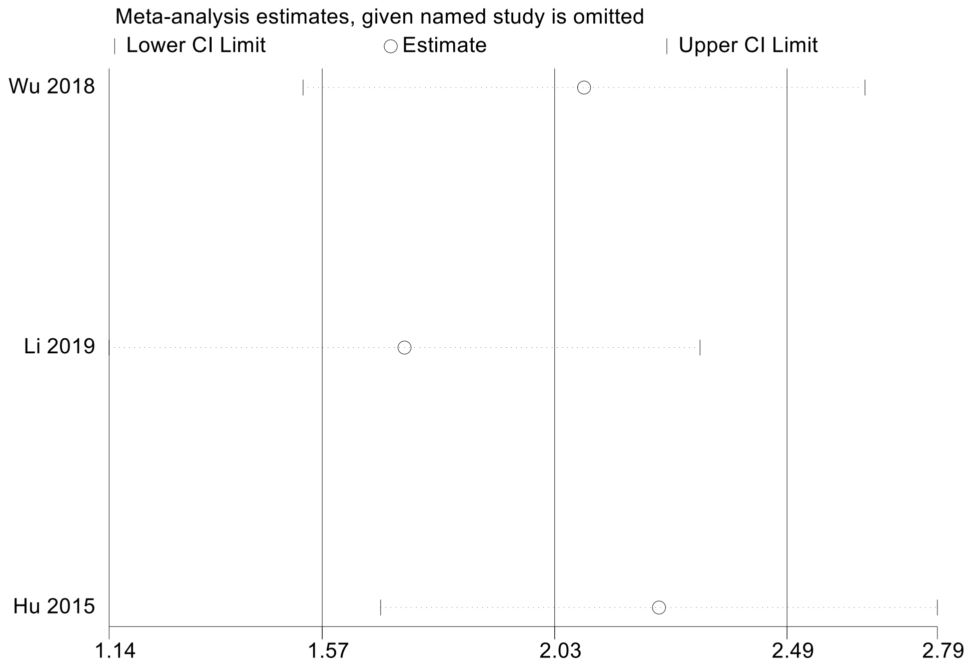

Exercise therapy

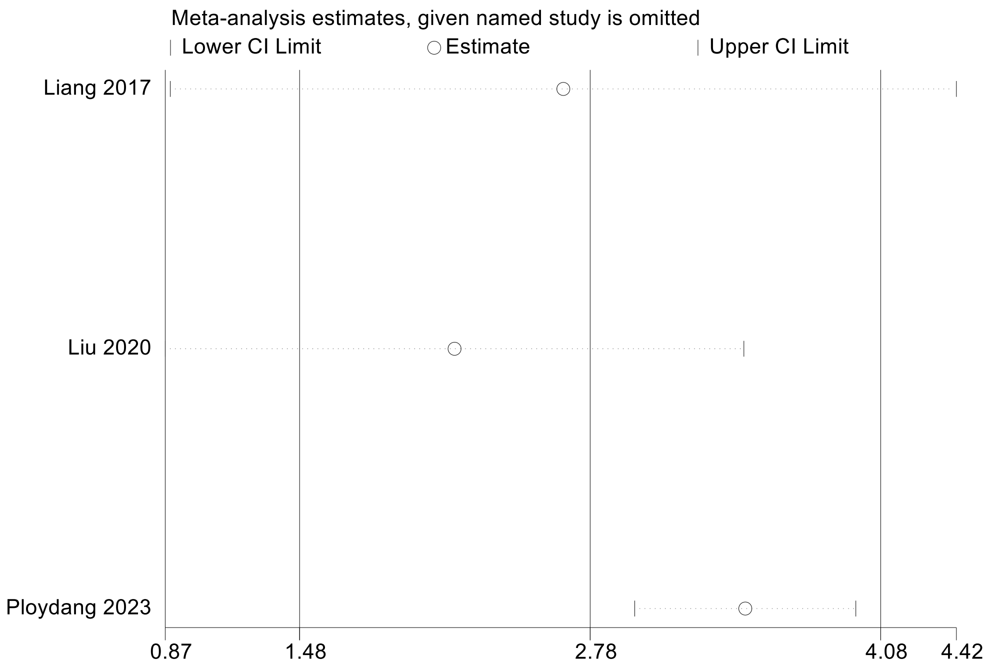

Meta-analysis estimates, given named study is omitted
Lower CI Limit
Estimate
Upper CI Limit
Liang 2017
Liu 2020
Ploydang 2023
0.87
1.48
2.78
4.08
4.42
