# Supplementary material for: Effects of non-pharmacological interventions on cognitive function in patients with type 2 diabetes mellitus and mild cognitive impairment: A network meta-analysis
Source: PLoS One. 2025 Aug 12;20(8):e0329397. doi: 10.1371/journal.pone.0329397 (PMC12342316; doi:10.1371/journal.pone.0329397)
Supplement: S2 Fig — (PDF) [file pone.0329397.s008.pdf]

1. Cognitive training VS Usual care

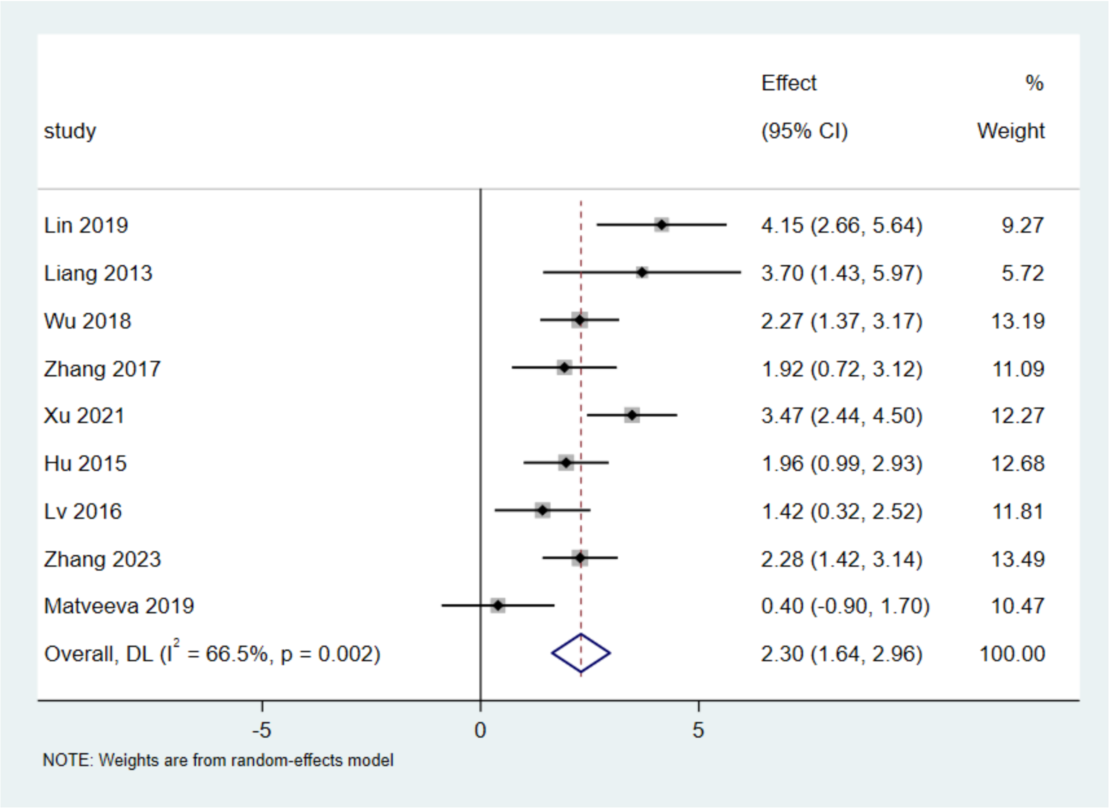

2. Exercise therapy VS Usual care

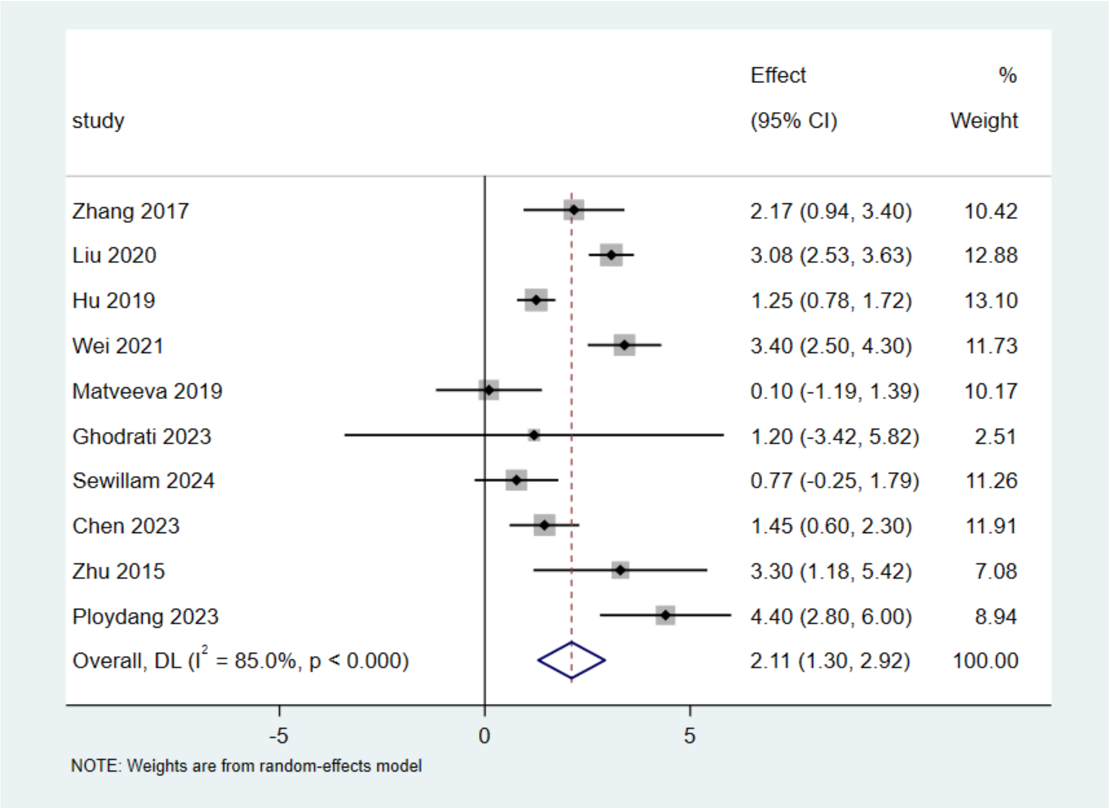

3. TCM therapy VS Usual care

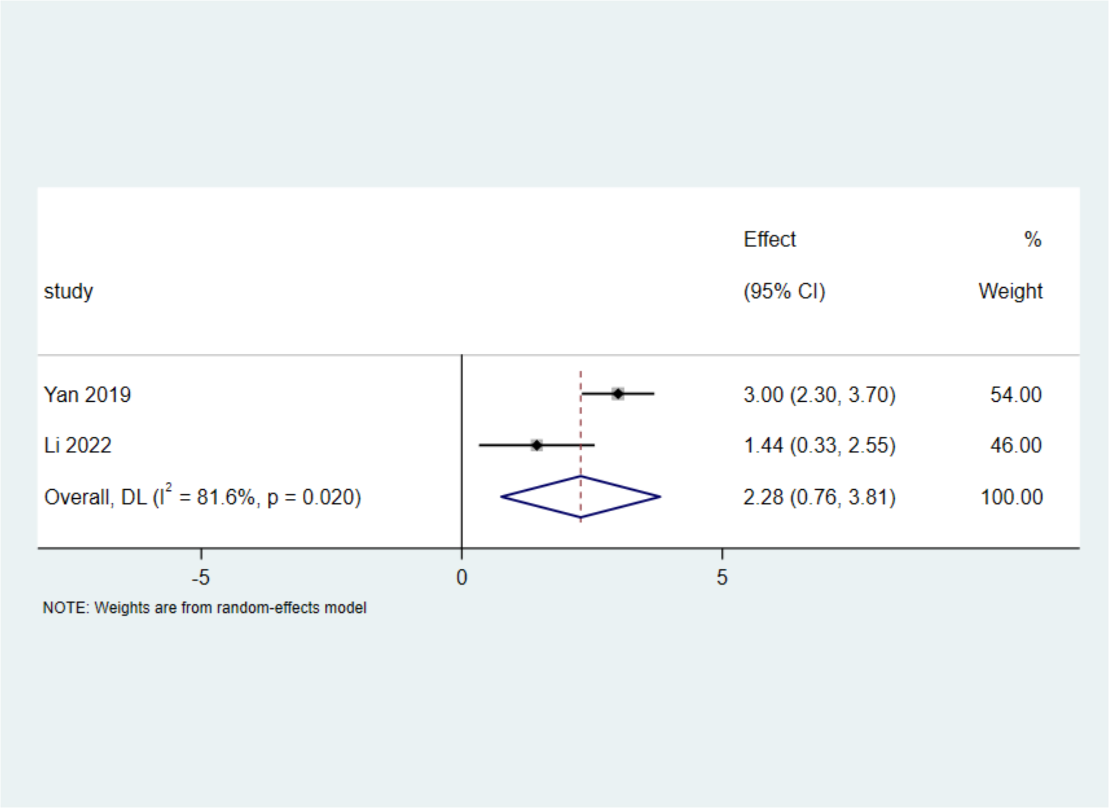

4. Comprehensive intervention VS Usual care

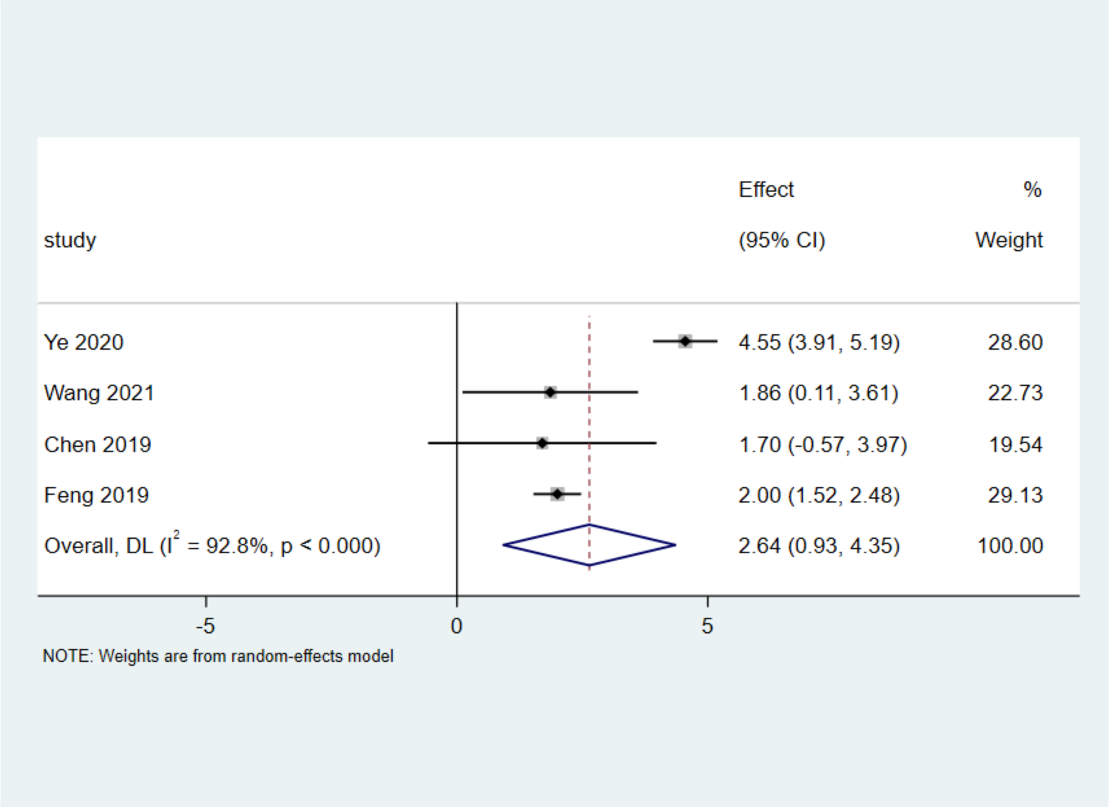

Comprehensive intervention VS Usual care (after excluding studies by Ye 2020)

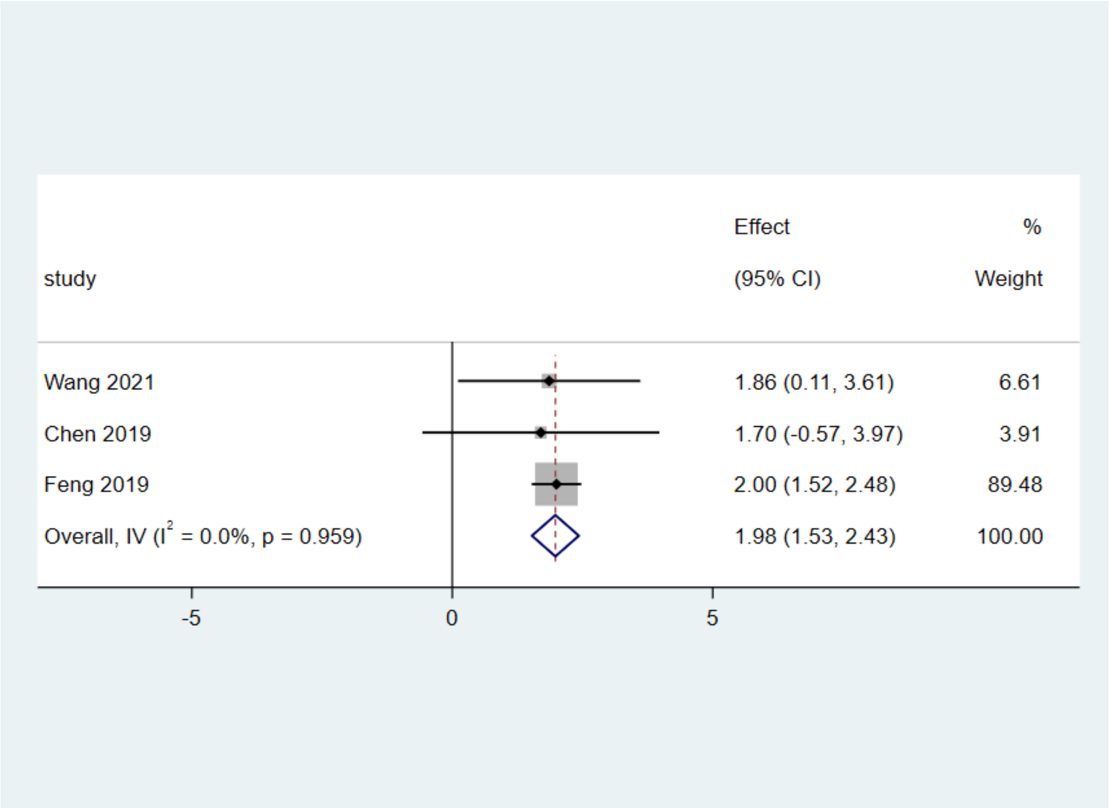

Effect
%
study
(95% CI)
Weight
Wang 2021
1.86 (0.11, 3.61)
6.61
Chen 2019
1.70 (-0.57, 3.97)
3.91
Feng 2019
2.00 (1.52, 2.48)
89.48
Overall, IV (I2 = 0.0%, p = 0.959)
1.98 (1.53, 2.43)
100.00
-5
0
5
