# Supplementary material for: Effects of non-pharmacological interventions on cognitive function in patients with type 2 diabetes mellitus and mild cognitive impairment: A network meta-analysis
Source: PLoS One. 2025 Aug 12;20(8):e0329397. doi: 10.1371/journal.pone.0329397 (PMC12342316; doi:10.1371/journal.pone.0329397)
Supplement: S3 Fig — (PDF) [file pone.0329397.s009.pdf]

1. Cognitive training VS Usual care

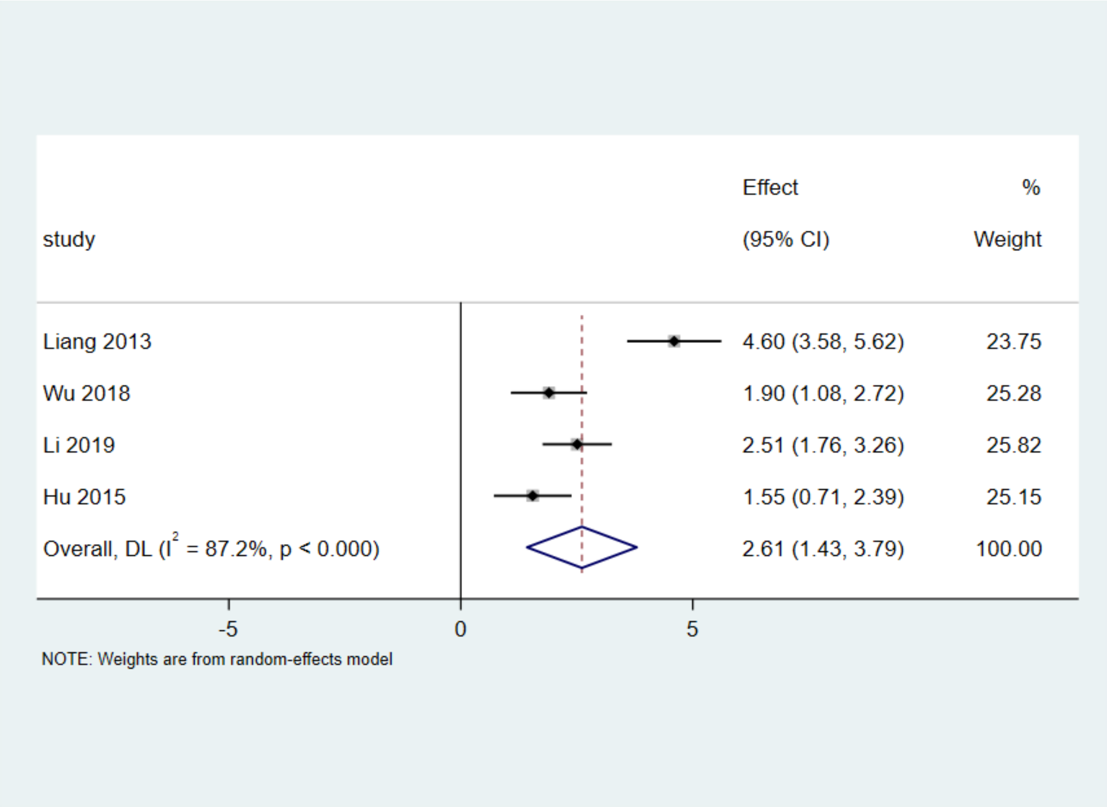

Cognitive training VS Usual care (after excluding studies by Liang 2013)

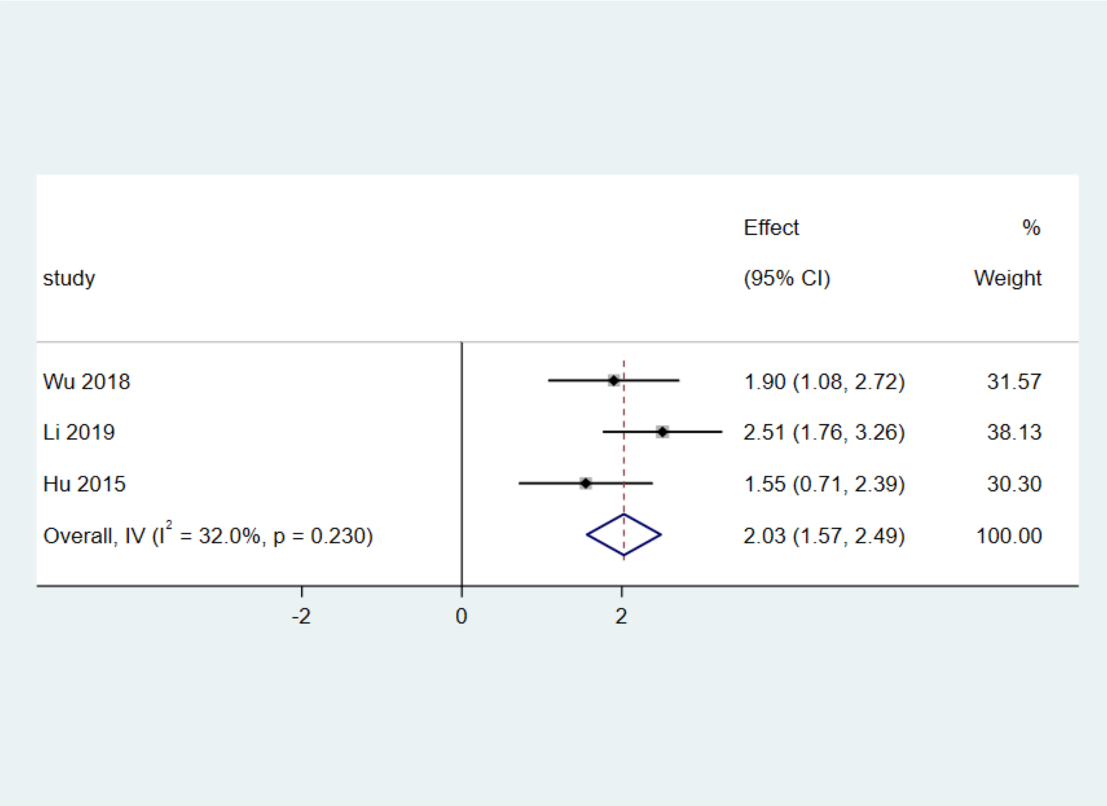

2. Exercise therapy VS Usual care

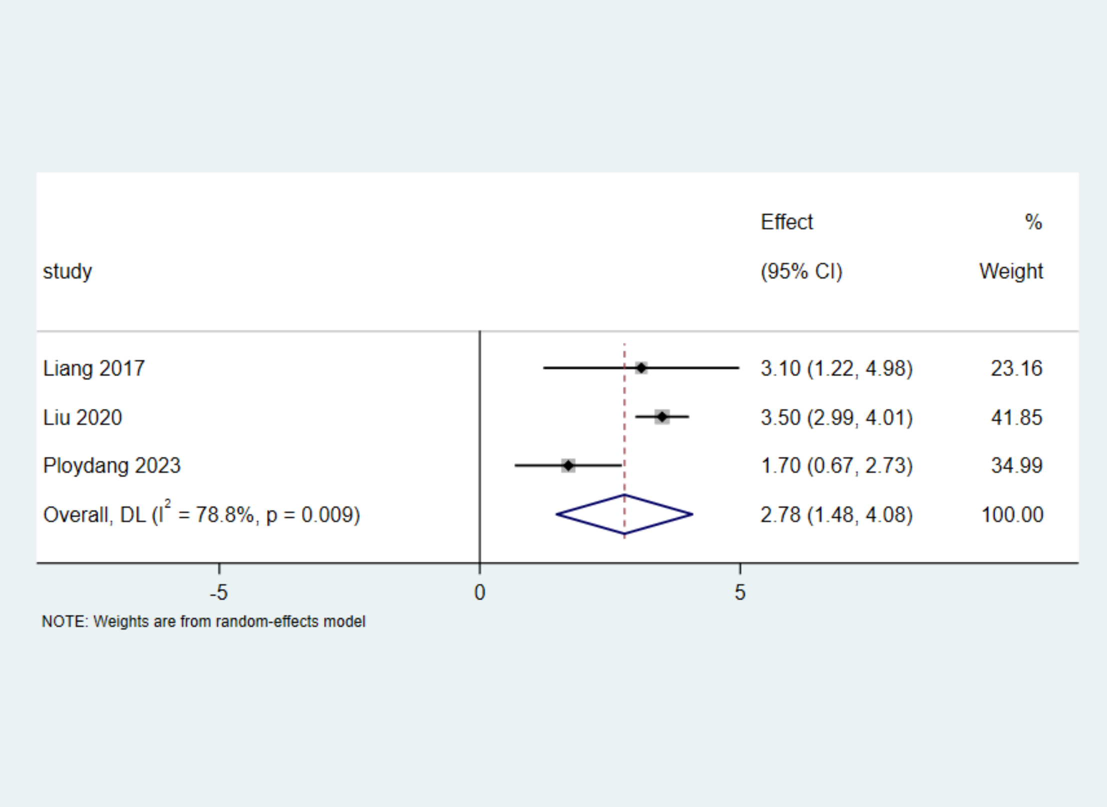

3. TCM therapies VS Usual care

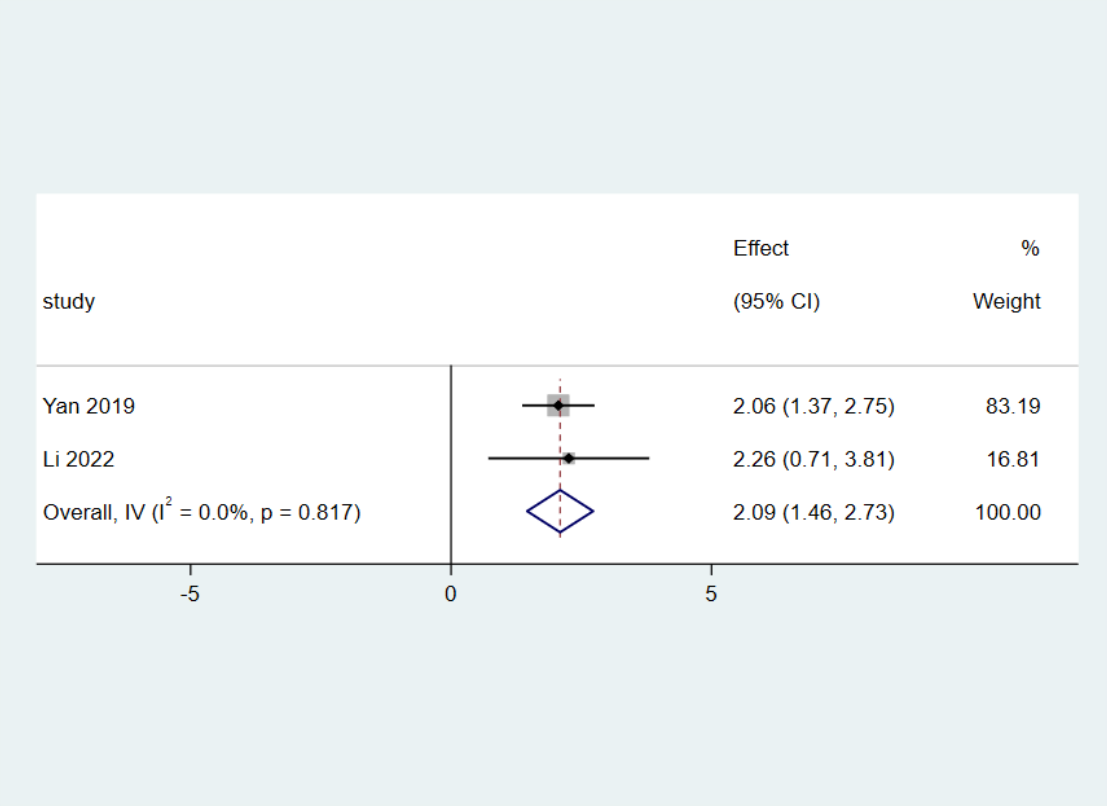

4. Comprehensive intervention VS Usual care

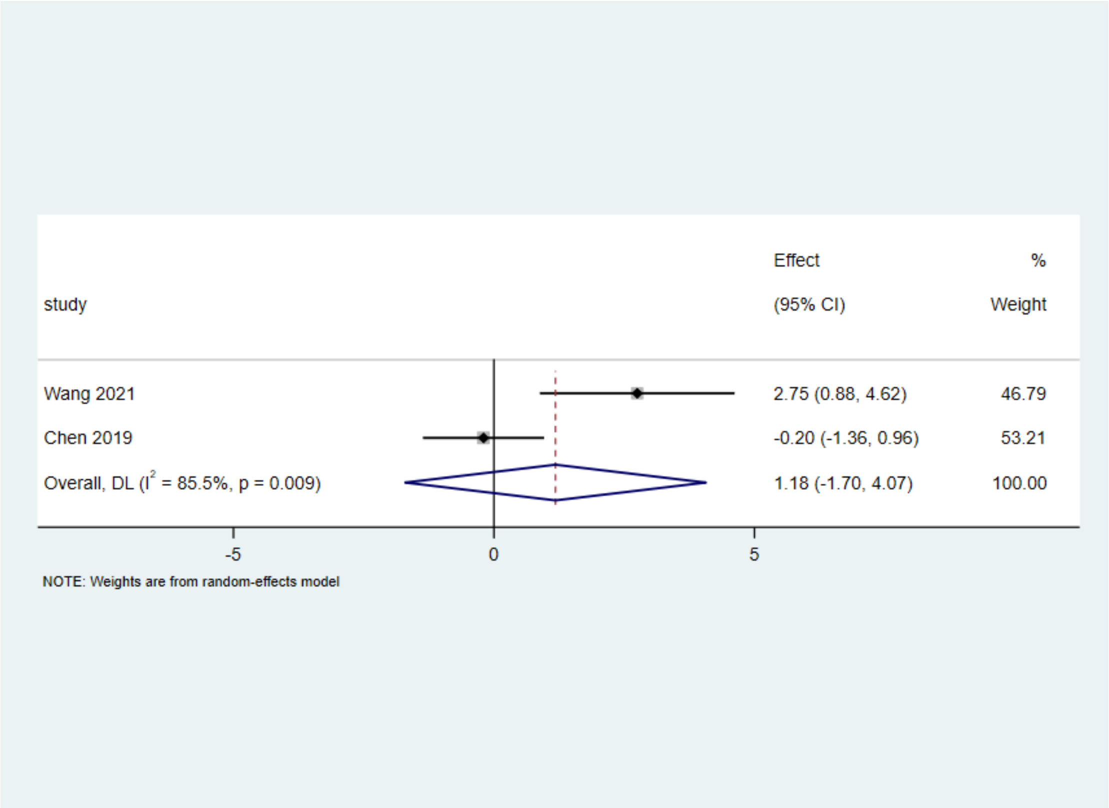

study
Effect
(95% CI)
%
Weight
Wang 2021
2.75 (0.88, 4.62)
46.79
Chen 2019
-0.20 (-1.36, 0.96)
53.21
Overall, DL (I² = 85.5%, p = 0.009)
1.18 (-1.70, 4.07)
100.00
-5
0
5
NOTE: Weights are from random-effects model
