# Supplementary material for: Effects of non-pharmacological interventions on cognitive function in patients with type 2 diabetes mellitus and mild cognitive impairment: A network meta-analysis
Source: PLoS One. 2025 Aug 12;20(8):e0329397. doi: 10.1371/journal.pone.0329397 (PMC12342316; doi:10.1371/journal.pone.0329397)
Supplement: Attachment — Submitted filename: Review Comments to the Author.docx [file pone.0329397.s011.docx]

**Review Comments to the Author**

**General Comments:** This manuscript explores the impact of non-pharmacological interventions on cognitive function in patients with Type 2 Diabetes Mellitus (T2DM) and mild cognitive impairment (MCI). The study addresses a clinically significant and underexplored topic, providing valuable insights for researchers and clinicians. The use of network meta-analysis enhances the methodology, and the inclusion of multiple interventions broadens the perspective on therapeutic options. However, some areas need further clarification and improvements to meet journal standards.

**Major Suggestions for Improvement:**

1. **Clarity in Results Section:**
   - Provide more detailed comparisons of the effectiveness of each intervention. For example, how does traditional Chinese medicine therapy compare quantitatively to cognitive training or exercise therapy regarding MoCA and MMSE scores?
   - Highlight key statistical values (e.g., p-values, confidence intervals) in the discussion for better clarity.
2. **Depth of Analysis:**
   - Expand on the neurobiological mechanisms underlying cognitive improvements seen with interventions like exercise therapy and traditional Chinese medicine. Include details on the roles of growth factors and inflammatory cytokines, which are critical to understanding the underlying mechanisms.
3. **Addressing Limitations:**
   - Elaborate on methodological limitations, particularly biases due to the lack of blinding and allocation concealment in some studies. Addressing this will increase the manuscript's credibility.
   - Discuss how the small number of included studies may impact the generalizability of the findings and propose strategies to address these gaps in future research.
4. **Consistency and Terminology:**
   - Ensure consistent use of key terms, such as “MCI,” “cognitive training,” and “traditional Chinese medicine therapy,” throughout the manuscript to improve readability.
   - Simplify complex sentences in the discussion to make the manuscript more accessible to a broader audience.
5. **Future Directions:**
   - Recommend specific areas for future research, such as comparative studies between acupoint massage and acupuncture or long-term follow-up studies to assess the sustainability of the benefits.

**Major Points:**

1. **Data Availability:**
   - It is unclear whether all data underlying the findings have been made fully available, as required by PLOS ONE’s data policy. Please ensure that raw data, such as individual data points behind summary statistics, are provided or deposited in a public repository. If restrictions apply, these should be clearly explained in the manuscript.
2. **Results and Clarity:**
   - The results section could benefit from more detailed comparisons of interventions, particularly between cognitive training, exercise therapy, and traditional Chinese medicine therapy. Highlighting specific statistical values in the discussion will improve clarity and support your conclusions.
   - Consider adding visual aids, such as summary charts or network diagrams, to improve the reader’s understanding of key findings.
3. **Methodological Transparency:**
   - High heterogeneity (e.g., I² > 80%) was noted in several analyses. While random-effects models were used appropriately, a more detailed exploration of potential sources of heterogeneity is necessary.
   - Some included studies lacked blinding or allocation concealment. Please address how these limitations might affect the reliability of your conclusions and discuss strategies to mitigate these issues in future research.
4. **Language and Readability:**
   - While the manuscript is written in standard English, certain sections, particularly the discussion, contain overly complex sentences. Simplifying these would enhance readability.
   - Ensure consistent terminology throughout the manuscript (e.g., "traditional Chinese medicine therapy" vs. "TCM therapies").
5. **Figures and Tables:**
   - Improve the legends for figures and tables to provide more detailed explanations of the data presented. Consider using comparative charts to summarize the effectiveness of interventions (e.g., SUCRA rankings).

**Minor Points:**

1. **Formatting and Compliance:**
   - Adhere strictly to PLOS ONE’s formatting guidelines, including consistent citation formatting and a clear structure for supplementary materials.
2. **Discussion:**
   - Expand the discussion on the practical applications of your findings and suggest specific directions for future research, such as evaluating long-term effects or combining interventions.

**Conclusion:** This manuscript has the potential to make a significant contribution to the field. However, revisions are necessary to address data availability, methodological transparency, and clarity in the presentation of the results. These improvements will strengthen the manuscript’s scientific rigor and impact.
