# Supplementary material for: Effects of non-pharmacological interventions on cognitive function in patients with type 2 diabetes mellitus and mild cognitive impairment: A network meta-analysis
Source: PLoS One. 2025 Aug 12;20(8):e0329397. doi: 10.1371/journal.pone.0329397 (PMC12342316; doi:10.1371/journal.pone.0329397)
Supplement: Attachment — Submitted filename: Response to Reviewers.docx [file pone.0329397.s014.docx]

**Response Letter**

Dear editors and reviewers,

We are very grateful for your constructive comments and suggestions for our manuscripts entitled “Effects of non-pharmacological interventions on cognitive function in patients with type 2 diabetes mellitus and mild cognitive impairment: a network Meta-analysis” (ID: PONE-D-24-54429). Your comments are very valuable and helpful for improving our manuscripts.in the following, the responses to all the comment are provided one by one.

We have tried our best to make all the revisions clear, and we hope that the revised manuscript can satisfy the requirements for publication.

The main revisions in the new manuscript are:

**Reply to the editor’s suggestions for article modification**

1. This study was modified according to the format template for viewing PLOS ONE, which meets the format requirements of PLOS ONE.
2. We have registered an ORCID iD: 0009-0004-4777-6524.
3. We have explained in the main text and Supplementary Information S2 Table the reasons for excluding each study after reading the full text. S2 Table also provides the first author, year and title of each excluded study. All the included and excluded literatures do not include unpublished studies.
4. We included the name of the literature manager and the date of data extraction. We extracted the data using EndNote and placed the extracted data in the Supplementary Information, S1 File. And it provided the main result data of paired Meta-analysis, as well as the league table of network Meta-analysis and the SUCRA values of each intervention measure.
5. We supplemented a complete risk assessment table for each item included in the study, which was made using Revman and placed in Fig 2 and Supplementary Information S1 Fig.

**Reply to the reviewer's suggestions for article modification**

1. **Clarity in Results Section:** In the manuscript, we have described the quantity of each intervention measure. In the abstract, results and discussion sections, we have highlighted the key statistical values to enhance the clarity of the results.
2. **Depth of Analysis:** Due to the abundance of content in the discussion, we have added subheadings to enhance clarity. We have delved deeply into the neurobiological mechanisms by which exercise therapy and traditional Chinese medicine can improve the cognitive function of patients with T2DM and MCI. In the exercise therapy, we have provided an in-depth description of the mechanisms of action of relevant growth factors and inflammatory cytokines. Since the main traditional Chinese medicine therapies included in this study are acupressure and acupuncture, we have explored the mechanisms of these two therapies. See page 21-23 of the manuscript for details.
3. **Addressing Limitations:** We conducted a thorough analysis of the limitations of the methodology, and discussed the reasons and countermeasures for the biases caused by the few included literatures, the lack of blinding and allocation concealment in some studies. See page 23 of the manuscript for details.
4. **Consistency and Terminology:** We have read through the entire manuscript and unified the key terms. For instance, we will define the traditional Chinese medical therapy that appears for the first time in the text as TCM therapy, and henceforth it will be referred to as such throughout the text. We also simplified the more complex sentences in the original discussion section, especially in the part that describes the mechanism of action of acupoint massage in traditional Chinese medicine. Some professional terms used in traditional Chinese medicine might be rather obscure and difficult to understand. We optimized them to make the manuscript more accessible to a wider range of readers. See page 22 of the manuscript for details.
5. **Future Directions:** We have delineated the directions for future clinical practice and research in this field, including standardizing operation procedures, conducting long-term follow-ups, comprehensively evaluating outcome indicators, and adopting new technologies to enhance patient compliance. Additionally, we have proposed that more high-quality research should be conducted in the future to further explore the efficacy, patient acceptance, and mechanism of action between acupoint massage and acupuncture. See page 24 of the manuscript for details.

**Reply to points**

1. **Data Availability:** All relevant data are within the manuscript and its appendix files.
2. **Results and Clarity:** We have respectively provided explanations in the text regarding the number of included literature for each intervention measure in each outcome indicator. Specific statistical values have been highlighted in the abstract, results and discussion sections of the manuscript.
3. **Methodological Transparency:** Regarding the high heterogeneity observed in the pairwise Meta-analysis results, we conducted sensitivity analyses for each intervention measure in each of the two outcome indicators separately. The results indicated that: In the comprehensive intervention where MoCA score was used as the outcome indicator, after eliminating the study by Ye et al., the heterogeneity decreased. The analysis suggests that this might be due to the fact that in the comprehensive intervention measures, the cognitive training component of this study accounted for a relatively large proportion while other intervention measures were relatively less prominent. In the cognitive training with MMSE score as the outcome indicator, after eliminating the study by Liang et al., the heterogeneity decreased. The analysis might be attributed to the relatively short duration of the intervention in this study. There was no significant change in the heterogeneity of other intervention measures. The Meta-analysis results were robust. The results of the sensitivity analysis can be found in Supplementary Information S2 Fig. The heterogeneity of the network meta-analysis is relatively small.
   We conducted a thorough analysis of the limitations of the methodology, and discussed the reasons and countermeasures for the biases caused by the few included literatures, the lack of blinding and allocation concealment in some studies. See page 23 of the manuscript for details.
4. **Language and Readability:** We have made partial revisions to overly complex sentences and checked the entire manuscript to ensure consistency in terminology.
5. **Figures and Tables:** We plotted cumulative probability ranking diagrams (Fig 4 and Fig 5) for the two outcome indicators of different intervention measures, and sorted the intervention measures based on the SUCRA values. The detailed tables can be found in Supplementary Information S3 Table.
6. **Formatting and Compliance:** This study strictly adhered to the format guidelines of PLOS ONE and revised the citation format and supplementary information.
7. **Discussion:** We have expanded the discussion section, pointed out the directions for future clinical practice and scientific research, and proposed that personalized comprehensive intervention measures should be carried out based on the specific conditions of patients and long-term follow-up should be conducted to verify the effectiveness and safety of the measures, with the aim of better improving the cognitive function of patients with T2DM and MCI and enhancing their quality of life. For details, please refer to page 24 of the manuscript.

Sincerely,

Corresponding author.
