# Supplementary material for: Effects of non-pharmacological interventions on cognitive function in patients with type 2 diabetes mellitus and mild cognitive impairment: A network meta-analysis
Source: PLoS One. 2025 Aug 12;20(8):e0329397. doi: 10.1371/journal.pone.0329397 (PMC12342316; doi:10.1371/journal.pone.0329397)
Supplement: Attachment — Submitted filename: Response to the review of manuscript number PONE-D-24-54429.docx [file pone.0329397.s013.docx]

1. The study addresses the urgent clinical issue of cognitive decline in patients with Type 2 Diabetes Mellitus (T2DM) and mild cognitive impairment (MCI)—conditions that are increasingly prevalent in aging populations.

2. The authors conducted a network meta-analysis of 25 randomized controlled trials involving 2,446 participants, using MoCA and MMSE as cognitive outcome indicators.

Their approach follows the PRISMA guidelines and includes sensitivity analysis, bias assessment, and SUCRA rankings, ensuring scientific rigor and transparency.

3. The findings highlight cognitive training and exercise therapy as the most effective non-pharmacological interventions, offering clinicians evidence-based alternatives to pharmacotherapy for T2DM patients with MCI.

4. The discussion integrates neurobiological mechanisms, such as the roles of BDNF, IGF-1, VEGF, and neuroinflammation, offering insight into why these interventions work, which strengthens the clinical relevance.

5. The authors outline specific recommendations for clinical practice, such as personalizing interventions and improving adherence.
