# Supplementary material for: Effects of non-pharmacological interventions on cognitive function in patients with type 2 diabetes mellitus and mild cognitive impairment: A network meta-analysis
Source: PLoS One. 2025 Aug 12;20(8):e0329397. doi: 10.1371/journal.pone.0329397 (PMC12342316; doi:10.1371/journal.pone.0329397)
Supplement: Attachment — Submitted filename: Response_to_Reviewers_auresp_2.docx [file pone.0329397.s015.docx]

**Response Letter**

Dear editors and reviewers,

We are very grateful for your constructive comments and suggestions for our manuscripts entitled “Effects of non-pharmacological interventions on cognitive function in patients with type 2 diabetes mellitus and mild cognitive impairment: a network Meta-analysis” (ID: PONE-D-24-54429). Your comments are very valuable and helpful for improving our manuscripts.in the following, the responses to all the comment are provided one by one.

We have tried our best to make all the revisions clear, and we hope that the revised manuscript can satisfy the requirements for publication.

**Reply to the editor’s suggestions for article modification**

1.We reviewed the reference list of this article to ensure that no citations were made to withdrawn papers. All the references have been revised in accordance with the formatting requirements of the PLOS ONE journal.

2.All the graphic files have been modified using the PACE digital diagnostic tool.

**Reply to the reviewer's suggestions for article modification**

*1.“Methods Sections 1.1–1.2 (Literature Search and Eligibility Criteria):*

*Provide detailed database search information (PubMed, Web of Science, Embase, Cochrane Library, CNKI, VIP, Wanfang, SionMed), including search dates and full search strings in the main text or appendix. Clearly list each inclusion and exclusion criterion (e.g., MCI diagnostic criteria, intervention definitions, outcome measures) and include a PRISMA flow diagram to ensure reproducibility. ”*

**Response**: In the article, we have specified the specific databases and the retrieval dates for the literature search, and provided detailed retrieval information for each database in Supplementary Information S1 Table. We clearly listed each inclusion and exclusion criterion, revised the MCI diagnostic criteria (please refer to page 7 of this manuscript), and clearly defined and cited relevant references based on the American College of Sports Medicine and previous studies regarding the specific definitions of various non-pharmacological intervention measures (please refer to page 7 of this manuscript). Regarding the outcome indicators, we further clarified the outcome indicators of this study and pointed out that the higher the scores of MoCA and MMSE tests, the better the cognitive function of the patients (please refer to page 8 of this manuscript). And we strictly followed the PRISMA guidelines to re-create the literature screening flowchart.

*2. “Methods Section 1.4 (Quality Assessment):
Specify the version of the risk‑of‑bias tool used (e.g., Cochrane RoB 2.0) and describe the criteria for judging each domain (random sequence generation, allocation concealment, blinding, data completeness, selective reporting, etc.). Present a summary table of risk‑of‑bias judgments (low/unclear/high) for all included studies rather than only a figure.”*

**Response**: We specified the version of the bias risk assessment tool used (RoB 2.0) and introduced six aspects for evaluating the quality of the literature. Each dimension was classified as "yes" (low risk), "no" (high risk), or "unclear" (moderate risk). We re-generated the quality evaluation chart for the included literature (Fig 2) based on RoB 2.0. The summary table for the assessment of bias risk for all included studies is presented in Supporting information S3 Table.

*3. “Methods Section 1.5 (Statistical Analysis):*

*Justify the choice of fixed‑effect versus random‑effect models (e.g., use random‑effect when I² ≥ 50%). Detail the methods for testing consistency (e.g., node‑splitting or design‑by‑treatment interaction models) and assessing heterogeneity (I², τ²). Specify the software and package versions (e.g., R version, Stata version, relevant R packages/functions).”*

**Response**: We specified the versions of the software and packages used for data analysis (Stata/SE 15.1 and R 4.3.1, with data analysis conducted through the gemtc package), and explained that the effect model was selected based on the size of heterogeneity *I^2^*. When *I^2^* was less than 50%, a fixed effect model was chosen; otherwise, a random effect model was used. If a closed loop was formed, the method of node splitting was employed for the inconsistency test.( please refer to page 9-10 of this manuscript)

*4. “Results Sections 2.4 (Pairwise Meta‑Analysis) & 2.5.2.3 (Network Meta‑Analysis Convergence and Sensitivity):*

*Incorporate sensitivity analyses for both pairwise and network meta‑analyses. Show forest plots or tables comparing heterogeneity statistics (I²) and effect estimates before and after excluding high‑risk studies or switching between fixed and random effects, and discuss the impact on conclusions.”*

**Response**: Our previous study had already conducted a sensitivity analysis for the pairwise Meta-analysis. The detailed results can be found in Supporting information S1 Fig. According to your suggestion, we conducted heterogeneity tests for the two outcome indicators in the network Meta-analysis and presented their respective *I^2^*, Dbar, pD and DIC values under the fixed effect model and the random effect model (on pages 16 and 18 of the manuscript). Due to the influence of the number of included studies, we conducted a sensitivity analysis for the included studies with MoCA score as the outcome indicator (on pages 19-20 of the manuscript). Unfortunately, we were unable to identify the source of heterogeneity. By excluding the study with the smallest sample size for the sensitivity analysis, we observed that the ranking of SUCRA did not change, indicating that our conclusion is relatively reliable. We have placed the results of the sensitivity analysis in Supporting information S5 Table, including the comparison of heterogeneity analysis data before and after the sensitivity analysis.

*5. “Results Section 2.5 (Sources of Heterogeneity and Subgroup/Meta‑Regression Analyses):*

*For comparisons with high I² (>50%), perform subgroup analyses or meta‑regression by intervention type (cognitive training, exercise, TCM, combined interventions), intervention duration (≤3 months vs. >3 months), baseline cognitive level, or patient age to explore potential moderators of effect.”*

**Response**: We conducted a Meta-regression using the outcome indicators of the MoCA score in the included studies. We analyzed the data by considering whether the intervention duration was > 3 months and whether the intervention frequency was > 3 times per week as covariates. However, we still failed to identify the source of heterogeneity. Therefore, we have to admit that the large heterogeneity is one of the limitations of this study. Detailed information can be found in Supporting information S5 table.

*6. “Results Section 2.5.2.2 (SUCRA Ranking Interpretation):*

*In the Results or Discussion, explain the meaning of SUCRA (0–100% scale, with higher values indicating a higher probability of being the best intervention) and relate these rankings to MD values and 95% CIs to guide interpretation of each intervention’s relative efficacy.”*

**Response**: In the 1.5 statistical methods section in this article, we explained the meaning of SUCRA, indicated that the range of SUCRA values is from 0 to 1, and stated that the larger the value, the better the effect of the intervention measure. We also provided explanations in the SUCRA ranking results of the two outcome indicators. At the same time, in the 1.5 statistical methods section, we explained the meaning of the MD value, stating that when the *MD* value is ≥ 0.8, it indicates a large effect size. In the results of the network Meta-analysis of the two outcome indicators, we respectively explained the *MD* values of the intervention measures. In the 3.1 main results section of the discussion part of this article, we combined SUCRA and *MD* values to explain the reliability of cognitive training as the best intervention measure for improving patients' cognitive function.

*7. “Discussion Section 3 (Clinical Implications):*

*Deepen the discussion of clinical relevance by comparing top‑ranked interventions (e.g., combined, cognitive training, exercise) with existing clinical guidelines or consensus statements for cognitive care in T2DM patients, highlighting which interventions are currently recommended or readily implementable, and offering practice recommendations based on your findings.”*

**Response**: Based on your suggestions, we extensively reviewed international guidelines and expert consensus, and combined existing clinical guidelines and expert opinions to explain several top-ranked non-pharmacological intervention measures (please refer to page 27 of this manuscript). Based on the above findings, we recommend that in clinical practice, cognitive training and exercise therapy be prioritized as basic and effective single intervention measures. When conditions permit, a combined comprehensive intervention combining the two should be given priority. Traditional Chinese medicine hospitals can also use traditional Chinese medicine treatments as supplementary or alternative therapies. At the same time, we have provided some specific suggestions for future clinical work for reference (please refer to page 28 of this manuscript).

*8. “Expand Discussion on Mechanisms and Clinical Implications:*

*Elaborate on the potential neurobiological mechanisms by which cognitive training, exercise, and TCM may improve cognition in T2DM + MCI patients. Discuss practical considerations for implementing these interventions in clinical settings and compare your results with existing meta-analyses in general MCI populations to highlight novel contributions.”*

**Response**: Based on your suggestions, we reorganized and elaborated in depth the potential neurobiological mechanisms by which cognitive training, exercise therapy and traditional Chinese medicine might improve the cognition of patients with T2DM and MCI. We discussed our ideas in clinical practice and compared our research with a previous meta-analysis on the cognitive function of MCI patients in the general population. The results were basically consistent. (See pages 22-25 and 28 of this manuscript.)

Sincerely,

Corresponding author.
